# Fentanyl effects on respiratory neuron activity in the dorsolateral pons

**DOI:** 10.1152/jn.00113.2022

**Published:** 2022-10-05

**Authors:** Sandy E. Saunders, David M. Baekey, Erica S. Levitt

**Affiliations:** ^1^Department of Pharmacology and Therapeutics, University of Florida, Gainesville, Florida; ^2^Center for Respiratory Research and Rehabilitation, University of Florida, Gainesville, Florida

**Keywords:** apnea, electrophysiology, Kölliker–Fuse, opioid, respiratory depression

## Abstract

Opioids suppress breathing through actions in the brainstem, including respiratory-related areas of the dorsolateral pons, which contain multiple phenotypes of respiratory patterned neurons. The discharge identity of dorsolateral pontine neurons that are impacted by opioids is unknown. To address this, single neuronal units were recorded in the dorsolateral pons of arterially perfused in situ rat preparations that were perfused with an apneic concentration of the opioid agonist fentanyl, followed by the opioid antagonist naloxone (NLX). Dorsolateral pontine neurons were categorized based on respiratory-associated discharge patterns, which were differentially affected by fentanyl. Inspiratory neurons and a subset of inspiratory/expiratory phase-spanning neurons were either silenced or had reduced firing frequency during fentanyl-induced apnea, which was reversed upon administration of naloxone. In contrast, the majority of expiratory neurons continued to fire tonically during fentanyl-induced apnea, albeit with reduced firing frequency. In addition, pontine late-inspiratory and postinspiratory neuronal activity were absent from apneustic-like breaths during the transition to fentanyl-induced apnea and the naloxone-mediated transition to recovery. Thus, opioid-induced deficits in respiratory patterning may occur due to reduced activity of pontine inspiratory neurons, whereas apnea occurs with loss of all phasic pontine activity and sustained tonic expiratory neuron activity.

**NEW & NOTEWORTHY** Opioids can suppress breathing via actions throughout the brainstem, including the dorsolateral pons. The respiratory phenotype of dorsolateral pontine neurons inhibited by opioids is unknown. Here, we describe the effect of the highly potent opioid fentanyl on the firing activity of these dorsolateral pontine neurons. Inspiratory neurons were largely silenced by fentanyl, whereas expiratory neurons were not. We provide a framework whereby this differential sensitivity to fentanyl can contribute to respiratory pattern deficits and apnea.

## Graphical abstract

**Figure:**
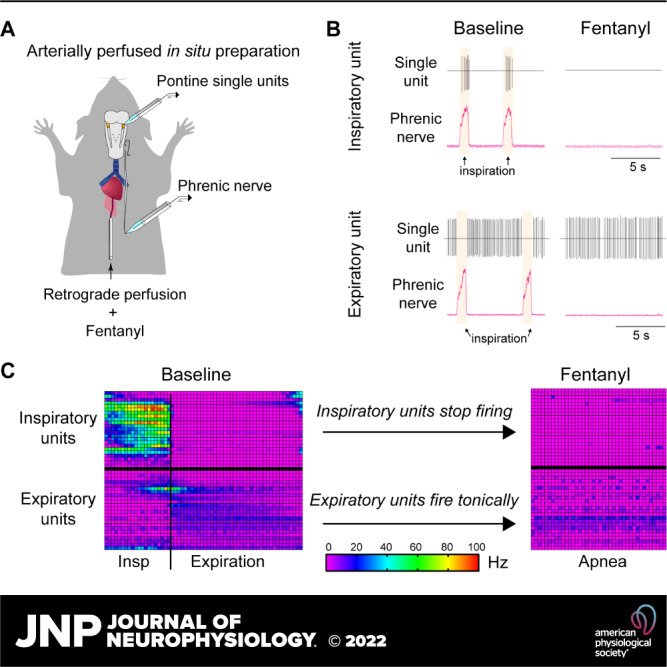

## INTRODUCTION

The proximal cause of death from opioids is inadequate ventilation via respiratory depression. At low doses, opioids increase tidal volume and slow respiratory frequency, which is sometimes useful in the treatment of dyspnea (1). As the opioid dose is increased, a reduction in tidal volume occurs (2). Although infrequently studied, rapid shallow breathing (tachypnea) may occur, temporarily maintaining ventilation and oxygenation (3, 4). As the opioid dose increases further, shallow breathing slows (bradypnea) and may stop all together (apnea) (5), leading to inadequate ventilation and death.

Opioid-induced respiratory depression is due to the activation of μ-opioid receptors (5, 6), which are located throughout the brainstem respiratory network (7–9). Recent work using genetic and pharmacological manipulations has determined that the dorsolateral pons, including the Kölliker–Fuse (KF) and lateral parabrachial complex, plays a key role in opioid-induced respiratory depression (5, 10–13). The dorsolateral pons is important for maintaining respiratory rate (11, 14–16) and producing a normal respiratory pattern (17–21). The dorsolateral pons also contains a significant population of neurons that are directly hyperpolarized by opioids (11). The discharge identity of medullary neurons expressing μ-opioid receptors is nondiscriminant, and includes preinspiratory, inspiratory, expiratory, and tonic neurons (22, 23). However, the discharge identity of dorsolateral pontine respiratory neurons affected by opioids remains unknown. To determine how dorsolateral pontine respiratory neuron activity is impacted by opioids, we used the in situ arterially perfused working heart-brainstem preparation of rats to simultaneously record extracellular dorsolateral pontine neuronal activity and respiratory motor neuron output (24, 25) following administration of an apneic concentration of fentanyl, and reversal with the opioid antagonist naloxone (NLX). This preparation maintains in vivo-like, three-phase (inspiration, postinspiration, and late-expiration) respiratory motor output in the absence of anesthesia and allowed us to investigate dorsolateral pontine neuronal activity during fictive fentanyl overdose consisting of persistent apnea without confounding peripheral and chemosensory factors that arise from the cessation of ventilation. The results show that the activity of pontine inspiratory units and a subset of inspiratory/expiratory phase-spanning units is lost during fictive fentanyl overdose, likely contributing to pontine-mediated opioid-induced respiratory deficits. This work provides insights into the role of the dorsolateral pons in opioid overdose and expands our understanding of the neuronal mechanisms underlying the deleterious effects of opioids.

## METHODS

### Animals

Rats (Sprague–Dawley; male and female; P26-32; Charles River Laboratories) were used for all experiments. Rats were group-housed on a 12-h light-dark cycle with ad libitum access to food and water at the University of Florida animal facility. Experiments were conducted in accordance with the National Institutes of Health and ARRIVE guidelines and with approval from the Institutional Animal Care and Use Committee of the University of Florida.

### Drugs

Fentanyl citrate was from Sigma-Aldrich (St Louis, MO), prepared as a stock solution (10 mM) in purified water, and administered to the perfusion reservoir (200 mL) for a final concentration of 300 nM. Naloxone was from Abcam (Cambridge, MA), prepared as a stock solution (10 mM) in purified water, and administered to the perfusion reservoir for a final concentration of 1 µM.

### In Situ Arterially Perfused Working Heart-Brainstem Preparation

Phrenic and vagus nerve recordings were obtained using the in situ arterially perfused working heart-brainstem preparations, as described previously (5, 11, 24). Rats were pretreated with heparin (1,000 U sc) 20 min before the start of surgical dissection. Rats were deeply anesthetized with isoflurane until visible respirations had nearly ceased. They were then bisected subdiaphragmatically, exsanguinated, and placed in ice-cold Ringers solution containing, in mM: 125 NaCl, 3 KCl, 2.5 CaCl_2_, 1.25 MgCl_2_, 1.25 KH_2_PO_4_, 10 d-glucose, and 24 NaHCO_3_. The occipital and parietal skull was removed, and aspiration was used to decerebrate rats at the precollicular level. The lungs were removed and the left phrenic nerve (PN) was isolated from the diaphragm and cut distally. The left central vagus nerve (cVN) was isolated from the neck and cut distally. The cerebellum was removed to allow access to the dorsal surface of the brainstem.

The preparation was placed in the recording chamber in a right lateral recumbent position, and a double lumen catheter was inserted in the descending aorta and perfused, via peristaltic pump (Watson-Marlow Pump Pro MPL 580; flow rate 21–24 mL·min^−1^), with warmed (32°C), carbogenated (95% O_2_/5% CO_2_), modified Ringer’s solution containing polyethylene glycol (Sigma-Aldrich, MW 20,000 1.25%) for oncotic pressure. The second lumen of the catheter output to a pressure transducer that allowed for monitoring and maintenance of perfusion pressure (50–70 mmHg). Vasopressin (200–400 pM, Sigma-Aldrich, St Louis, MO) was added to the perfusate to maintain vascular tone and perfusion pressure. The preparation was observed during recovery until respiratory-related movements became evident and vecuronium bromide (4 μg/mL, Patterson Veterinary, Greeley, CO) was then added to the perfusate to paralyze skeletal muscle. The preparation was then placed in a semiprone position and mounted on the ear bars of a stereotaxic frame (Kopf, Tujunga, CA).

The phrenic and vagus nerves were desheathed and whole nerve activity was recorded using glass suction electrodes. Recordings were AC amplified (A-M Systems Model 1700, A-M Systems, Carlsborg, WA), bandpass filtered (0.1–5 kHz), and digitized at 10 kHz (1401 A–D converter and Spike2 software, Cambridge Electronic Design, Cambridge, UK). Nerve activities were rectified and integrated (time constant = 50 ms).

In a separate set of experiments, tissue oxygen and pH were measured within the dorsolateral pons 2 mm ventral from the tissue surface using oxygen and pH microsensors (25 µm tip diameter) to verify that observed effects were drug related and not due to transient changes in pH or oxygen availability during exposure (Unisense, Aarhus, Denmark).

### Extracellular Recordings

Extracellular unit recording was achieved using a glass microelectrode fabricated from a borosilicate pipette on a Narishige PE-2 pipette puller. The microelectrode was filled with 1 M NaCl. Electrode impedance (∼14 MΩ) was intermittently measured in a brain slice with an Axopatch 1D amplifier under bridge mode. Using a micromanipulator (Kopf Model 1760), the microelectrode was inserted stereotaxically into the dorsolateral pons, perpendicular to the surface of the brainstem, 2.0 mm lateral to midline (obex), and 2 ± 1.0 mm below the surface of the brainstem. The caudal edge of the inferior colliculus and the remaining cerebellar lobes (post cerebellectomy) were used as an additional target for rostral/caudal positioning. Recordings were AC amplified, bandpass filtered (0.1–5 kHz), and digitized at 10 kHz. Units with apparent respiratory patterned discharge were recorded for ≥10 fictive breaths to establish a baseline, and then fentanyl (300 nM) was added to the perfusion solution. Once sustained apnea occurred (≥30 s), opioid antagonist naloxone (1 µM) was added to the perfusion solution to reverse opioid effects.

At the end of the experiment, the electrode was withdrawn from the tissue, the recording solution was replaced with fluorescent beads (FluoSpheres 580/605, 2% in saline, Invitrogen, Waltham, MA), the tip of the pipette was clipped with fine tweezers, re-zeroed, and reinserted into the recording site where 60–90 nL of fluorescent bead solution was injected. Following the experiment, the head was removed, fixed in paraformaldehyde (4%) for at least 24 h at 4°C and stored in PBS (4°C) until further processing. The brainstem was removed and coronal slices (100 μm) were cut in PBS using a vibratome (Leica VT1200S). Bright-field and fluorescent images (Nikon AZ100) were superimposed in Fiji (26) for injection site identification. The center of the injection site was then mapped on semi-schematic drawings of coronal slices through rat dorsolateral pons. The rat brain atlas (Paxinos and Watson, 2007) was used for reference.

### Spike Sorting

Offline spike sorting was performed using Spike 2 software with a combination of template matching and principal components analysis. Principal components analysis was performed using time as the *Z*-axis, which allowed for accurate interpretation of effects due to electrode drift and periods of inactivity during fentanyl administration. An interspike interval histogram for each sorted unit was generated in Spike 2 and used to confirm that the processed recordings represented a single neuron, i.e., all spikes had a refractory period greater than 2 ms. The resulting spike trains were exported into Excel for analysis of the neuron firing rate over standardized respiratory phases.

### Respiratory Unit Analysis

To characterize the neurons recorded, the respiratory cycle was divided into two phases—inspiration and expiration. The phase start and stop times were identified manually in Spike 2 and exported to Microsoft Excel. The beginning of the inspiratory phase was defined as the onset of phrenic nerve activity using the raw signal. The beginning of the expiratory phase was defined as the cessation of phrenic nerve activity also using the raw signal. The spike train of each unit was segregated into individual phases, manually in Excel, using phase start and stop times. Once segregated, the spike trains were normalized from phase start to stop. For each unit, normalized spike trains were binned into corresponding phase fractions using the Frequency function in Excel. Inspiration was divided into 20 consecutive bins (each bin being 5% of the corresponding inspiratory cycle, bin No. 1–20) with early-inspiration being defined as the first half (bins 1–10) and late-inspiration as the second half of inspiration (bin No. 11–20). Expiration was divided into 40 consecutive bins (each bin is 2.5% of the corresponding expiratory cycle, bin No. 21–60) with early-expiration being defined as the first half (bin No. 21–40) and late-expiration as the second half of expiration (bin No. 41–60). The number of bins/phases was chosen based on the fact that the expiratory duration (5 ± 2.7 s) was ≥2 times the inspiratory duration (0.9 ± 0.3 s) at baseline.

Once spike trains were binned appropriately, the number of spikes per bin was divided by bin duration (bin duration = phase duration/No. of bins) to determine the firing frequency (Hz) per bin. Due to the binning procedure, some expiratory phase bins with small duration and only a single action potential had an artificially high firing frequency. To mitigate these effects, breaths where the expiration duration was not ≥2 times the inspiration duration were cross-referenced with instantaneous firing frequency and adjusted accordingly. This mainly affected expiratory units in two-phase breaths after naloxone administration, where expiratory duration was short.

To group single neuronal units by discharge identity, baseline phase fraction histograms were created in GraphPad Prism for each unit from the average of 10 fictive baseline breaths. Neurons were placed into two main classes (inspiratory and expiratory) with subgroups, based on when the majority of discharge (>50% of activity) occurred. Overlapping histograms of mean unit activity over the respiratory cycle were used to manually “template match” units with similar firing patterns to form subgroups.

### Analysis of Fentanyl Effects on Unit Activity

Single-neuron recordings (*n* = 47) were analyzed during baseline (average of 10 breaths), fentanyl-induced apnea, and recovery with naloxone (average of 10 breaths). During fentanyl-induced apnea, a period equal to the expiratory duration of the last breath before apnea was sampled. The sampled period of apnea was selected to include peak activity during apnea, such that if a neuron fired even a single action potential during fentanyl-induced apnea, it was sampled. This was chosen to avoid over-exaggeration of opioids’ capacity to suppress neuronal activity. Phase fraction histograms were plotted as averages within each class or as heatmaps to display individual units using GraphPad Prism. In some experiments, phase fraction histograms were subtracted in Microsoft Excel in a bin-by-bin fashion to determine changes in neuronal activity relative to preceding phases, as indicated in results. The resulting heatmaps and histograms were exported into GraphPad Prism for further display and statistical analysis. In addition, the peak firing frequency [Hz, average (width 500 ms)] of each unit was determined during baseline (average peak among 10 breaths), fentanyl-induced apnea (peak during apnea), and after naloxone (average peak among 10 breaths).

In a subset of experiments (*n* = 29), pontine neuronal activity was analyzed during different transitional states of output (3-phase pattern, 2-phase pattern, and apnea) during fentanyl-mediated decline and naloxone-mediated recovery of respiratory motor output, as described in results. Phase fraction histograms were constructed for these transition states as described earlier.

### Statistical Analysis

Statistical comparisons were made using GraphPad Prism (v.9.1.1). All error bars represent means ± SE unless otherwise stated. Comparisons between two unpaired groups were made using unpaired *t* test. Comparisons between three or more unpaired groups were made using one-way ANOVA with Tukey’s post hoc test. Comparisons between three or more paired groups were made using repeated-measures one-way ANOVA with Tukey’s post hoc test. Phase fraction histograms of differences in firing frequency were analyzed using one-sample Wilcoxon signed-rank test.

## RESULTS

### Respiratory Motor Output during Baseline, Fentanyl, and Naloxone

**Figure 1.**
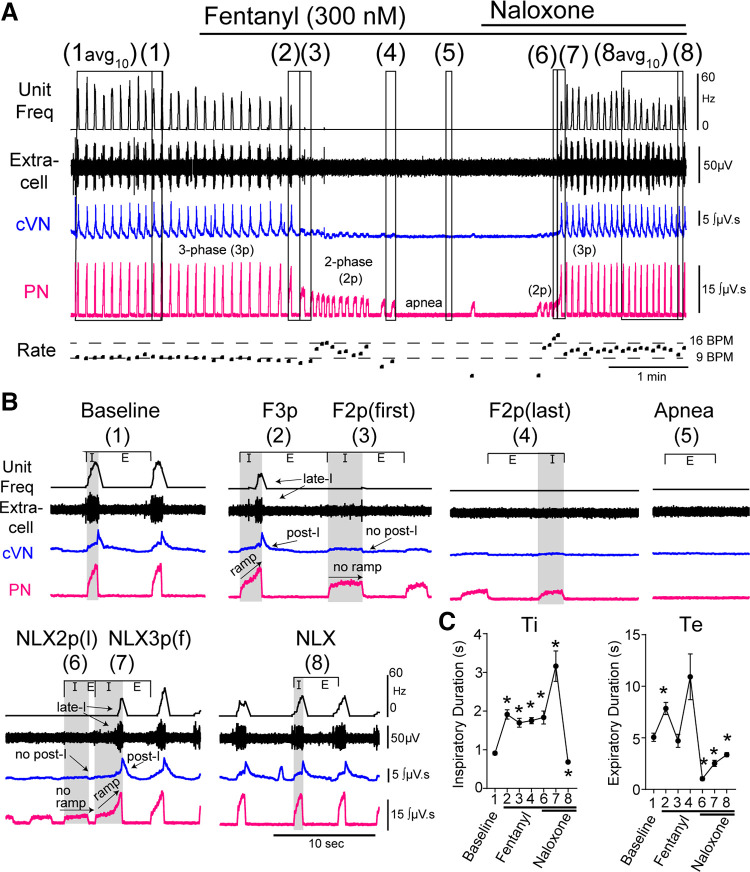
Respiratory motor output during fentanyl and naloxone (NLX) administration. Recordings from central vagus nerve (cVN), phrenic nerve (PN), and the dorsolateral pons were made using an in situ preparation of rat. *A*: continuous recording of (top to bottom) integrated instantaneous frequency of single dorsolateral pontine unit spiking activity (black, Unit Freq), raw signal from dorsolateral pontine extracellular recording (black, Extracell), integrated cVN activity (blue), and integrated PN activity (pink). Instantaneous respiratory rate [fictive breaths per minute (BPM)]) is shown below. Systemic application of fentanyl (300 nM) results in slowing of normal three-phase output, transition to low-amplitude, two-phase bursts, which then slowed resulting in sustained apnea. Systemic application of naloxone (1 μM) restores output in a similar but reversed manner to fentanyl-mediated decline. *B*: zoomed in view of epochs indicated in *A*. Avg_10_ is average of 10 breaths. Epochs are as follows: (1) baseline, (2) last three-phase breath in fentanyl (F3p), (3) first two-phase breath in fentanyl (F2p(f)), (4) last two-phase breath in fentanyl (F2p(l)), (5) Apnea, (6) last two-phase breath in naloxone (NLX2p(l)), (7) first three-phase breath in naloxone (NLX3p(f)), and (8) recovery after naloxone (NLX). *C*: inspiratory duration (Ti) and expiratory duration (Te) during each epoch. Symbols are group means ± SE, *n* = 47 rats. *P < 0.0001, compared with baseline by repeated-measures two-way ANOVA and Tukey’s post hoc test.

The arterially perfused in situ preparation of the rat was used to simultaneously record the spiking activity of single dorsolateral pontine neuronal units and respiratory motor output from phrenic and vagus nerves at baseline and during perfusion of the opioid agonist fentanyl and the opioid antagonist naloxone. The baseline respiratory patten of all preparations (*n* = 47) demonstrated ramping activity of the phrenic nerve and vagal activity with both inspiratory (I) and post-inspiratory components [Fig. 1*B*(1)]. Average respiratory rate during baseline conditions was 12 ± 5 breaths/min, and the expiratory duration (5.0 ± 2.7 s) was ≥2 times the inspiratory duration (0.9 ± 0.3 s) (Fig. 1*C*).

After a baseline recording period consisting of ≥10 fictive breaths, fentanyl (300 nM) was perfused. Fentanyl induced a decline in respiratory motor output from baseline to apnea that contained several intermediate steps (Fig. 1), as described previously (5). First, the baseline three-phase respiratory motor output slowed via increases in inspiratory and expiratory duration [Fig. 1 (*epoch 1* vs. *2*)]. Then, postinspiration was lost, and the respiratory motor output was shifted to a two-phase pattern. This two-phase pattern had a longer inspiratory duration than baseline and lacked ramping phrenic nerve activity and postinspiratory vagus nerve activity (Fig. 1, *B*and *C*, *epoch 3*). Next, the two-phase pattern slowed due to increases in expiratory duration (Fig. 1, *epoch 4*). Finally, sustained apnea occurred (Fig. 1, *epoch 5*).

Following sustained apnea ≥ 30 s, naloxone (1 µM) was perfused. Naloxone reversed apnea following a similar progression, but in the opposite order. First, a two-phase pattern emerged that contained fast, long-duration bursts (Fig. 1, *epoch 6*). The first three-phase breath after naloxone reversal had an especially long inspiratory duration as the ramping activity on the phrenic nerve and postinspiratory vagal nerve activity re-emerged (Fig. 1, *epoch 7*). Finally, a stable three-phase respiratory pattern was re-established that was faster than baseline due to shortened inspiratory and expiratory times (Fig. 1*C*, *epoch 8*).

In a subset (9 of 47) of preparations, motor output transitioned directly from three-phase pattern to apnea with no intermediate two-phase output. To properly assess these transition points in the output pattern, only preparations with ≥3 low amplitude two-phase breaths in the transition from the baseline to apnea (29 of 47) were included in the analysis of pattern transitions.

### Dorsolateral Pontine Neuronal Activity during Fentanyl-Induced Apnea and Naloxone Reversal

In 47 preparations, single-unit activity was recorded from the dorsolateral pons during baseline, fentanyl-induced apnea, and after recovery with naloxone.

#### Baseline.

**Figure 2.**
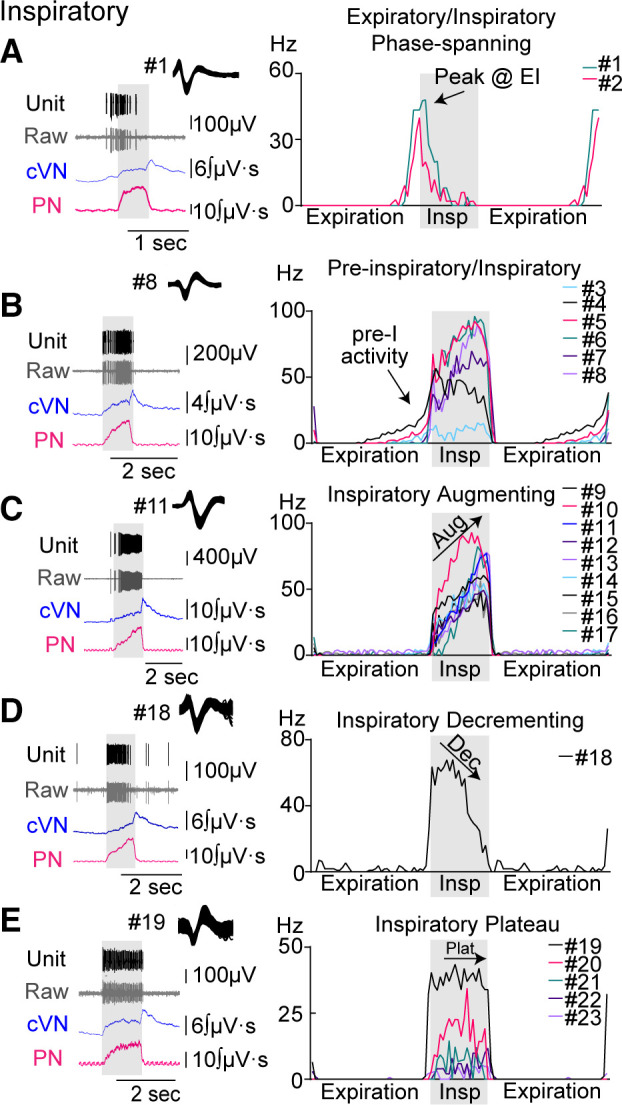
Discharge identity of dorsolateral pontine single units in the inspiratory class. Recordings from central vagus nerve (cVN, blue), phrenic nerve (PN, pink), and dorsolateral pontine units [raw data shown in gray (Extracell), sorted units shown in black (Unit)] were made using an in situ preparation of rat. Units were placed into subgroups depending on their pattern of activity over the respiratory cycle (*A*–*E*). *Left column*: example traces of a recorded unit with corresponding nerve output for each subgroup. *Right column* (histograms): average firing frequency per phase fraction bin for 10 consecutive breaths was plotted for individual units in each group. Each unit was assigned a number, which is indicated to the right of the histogram and is used consistently throughout the Figures. Inspiration (Insp) is indicated by gray shading. Expiration was duplicated to display phase transitions.

Neurons were classified into two main classes—inspiratory and expiratory—based on their spiking activity relative to phrenic nerve output during the baseline period. In the inspiratory class (*n* = 23, Fig. 2), five firing patterns were observed: expiratory/inspiratory phase-spanning (EIPS, 2 of 23, Fig. 2*A*), preinspiratory/inspiratory (pre-I/I, 6 of 23, Fig. 2*B*), inspiratory augmenting (I-aug, 9 of 23, Fig. 2*C*), inspiratory decrementing (I-dec, 1 of 23, Fig. 2*D*), and inspiratory plateau (I-plat, 5 of 23, Fig. 2*E*). EIPS neurons were placed in the inspiratory class because their preinspiratory activity overlapped closely with pre-I/I neurons.

**Figure 3.**
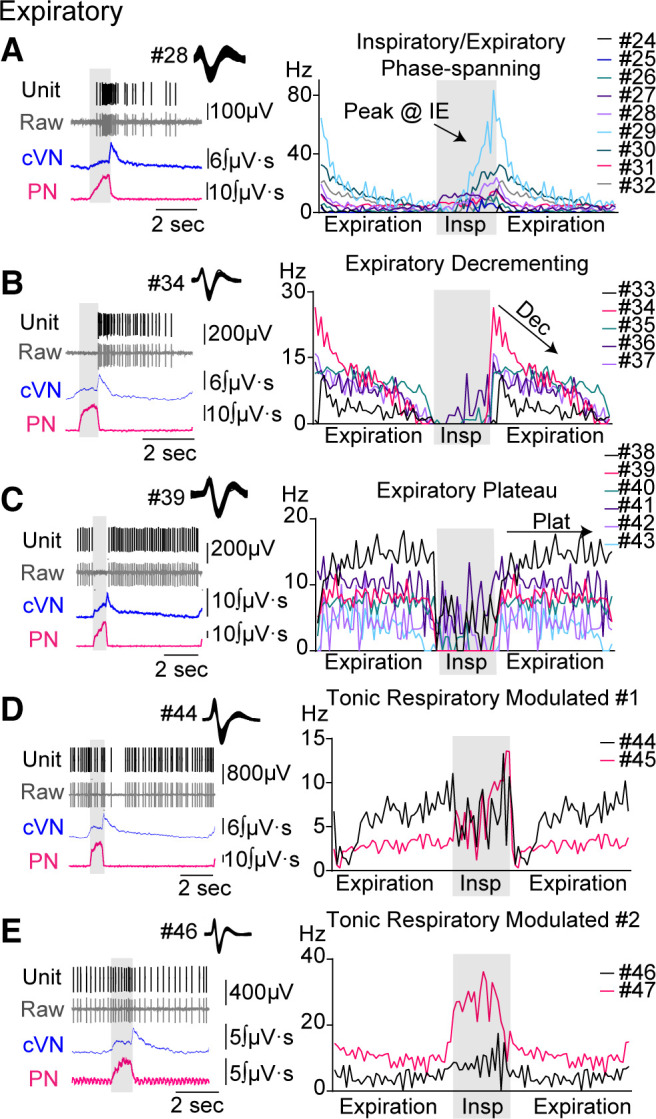
Discharge identity of dorsolateral pontine single units in the expiratory class. Recordings from central vagus nerve (cVN, blue), phrenic nerve (PN, pink), and dorsolateral pontine units [raw data shown in gray (Extracell), sorted units shown in black (Unit)] were made using an in situ preparation of rat. Units were placed into subgroups depending on their pattern of activity over the respiratory cycle (*A*–*E*). *Left column*: example traces of a recorded unit with corresponding nerve output for each subgroup. *Right column* (histograms): average firing frequency per phase fraction bin for 10 consecutive breaths was plotted for individual units in each group. Each unit was assigned a number, which is indicated to the right of the histogram and is used consistently throughout the Figures. Inspiration (Insp) is indicated by gray shading. Expiration was duplicated to display phase transitions.

In the expiratory class (*n* = 24, Fig. 3), five firing patterns were observed: inspiratory/expiratory phase-spanning (IEPS, 9 of 24, Fig. 3*A*), expiratory decrementing (E-dec, 5 of 24, Fig. 3*B*), expiratory plateau (E-plat, 6 of 24, Fig. 3*C*), tonic respiratory modulated expressing decreased activity during postinspiration (TRM1, 2 of 24, Fig. 3*D*), and tonic respiratory modulated expressing an increase in activity at inspiratory/expiratory phase transition (TRM2, 2 of 24, Fig. 3*E*). IEPS neurons were placed in the expiratory class because their postinspiratory activity was similar to E-dec neurons. Both groups of TRM neurons were placed in the expiratory class because >50% of activity occurred during expiration.

**Figure 4.**
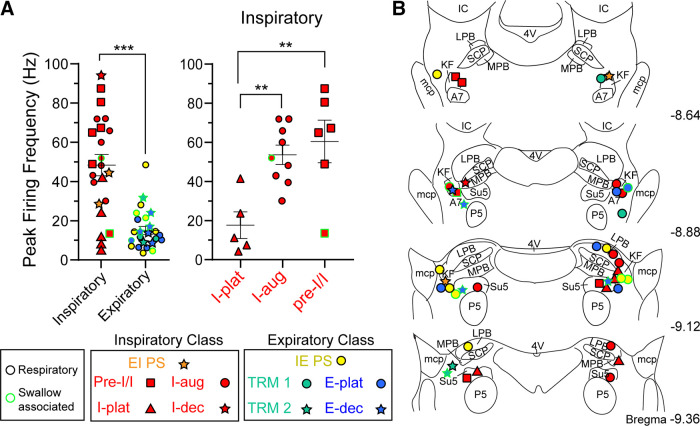
Baseline firing frequency and location of dorsolateral pontine units. *A*: summary of firing frequency of dorsolateral pontine single units at baseline. Data are grouped by class (inspiratory or expiratory) (*left*) or inspiratory subgroup (*right*). Symbols represent the average peak firing frequency over 10 consecutive breaths for individual units. Line and error are means ± SE. **P < 0.01, ***P < 0.001 by unpaired *t* test (*left*) or one-way ANOVA and Tukey’s post hoc test (*right*). Symbols are keyed for subclass (see results). Symbol outline represents if the unit had activity during only respiration (respiratory, black) or had activity during respiration and “swallowing” (multifunctional, neon green). *B*: semi-schematic drawings of coronal slices through rat dorsolateral pons. Location of recorded units was identified post hoc in coronal slices (*n* = 42 rats). A7, noradrenergic neurons; E-dec, expiratory decrementing; E-plat, expiratory plateau; EIPS, expiratory/inspiratory phase-spanning; I-aug, inspiratory augmenting; I-dec, inspiratory decrementing; I-plat, inspiratory plateau; IC, inferior colliculus; IEPS, inspiratory/expiratory phase-spanning; KF, Kölliker–Fuse; LPB, lateral parabrachial area; mcp, middle cerebellar peduncle; MPB, medial parabrachial area; pre-I/I, preinspiratory/inspiratory; SCP, superior cerebellar peduncle; TRM1, tonic respiratory modulated expressing decreased activity during postinspiration; TRM2, tonic respiratory modulated expressing an increase in activity at inspiratory/expiratory phase transition; 4V, fourth ventricle.

Firing frequency at baseline differed between and within classes (Fig. 4*A*). On average, the inspiratory class fired significantly faster than the expiratory class (mean difference: 32.3 ± 5.7 Hz, *P* < 0.0001). Among the inspiratory class, I-plat neurons fired slower at baseline than I-aug and pre-I/I neurons (mean difference between I-aug and I-plat: 36.0 ± 10.7 Hz, *P* = 0.0096; mean difference between pre-I/I and I-plat: 42.8 ± 11.6 Hz, *P* = 0.0049).

#### Fentanyl-induced apnea.

After the baseline period, fentanyl (300 nM) was added to the perfusate. In all 47 experiments, sustained apnea occurred after fentanyl administration (Fig. 1), as described previously (5).

**Figure 5.**
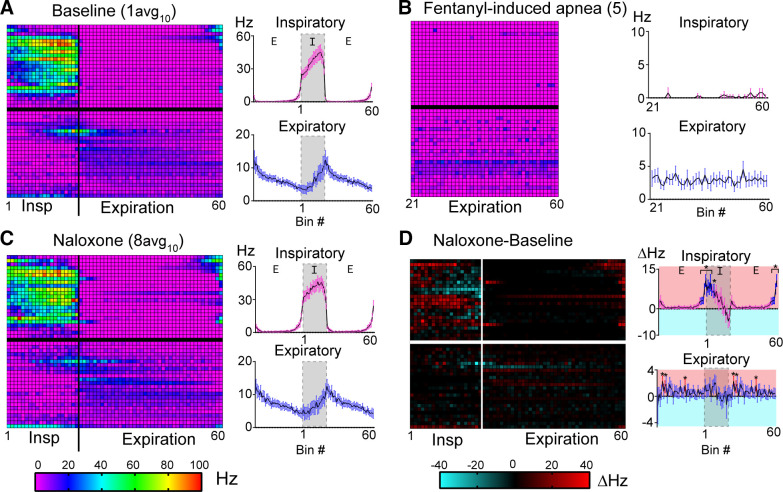
Phasic activity of dorsolateral pontine single units during baseline, fentanyl-induced apnea, and naloxone recovery. *A*–*C*, *left*: phase fraction two-dimensional (2-D) histogram (heatmap) displays activity of individual recorded units (*n* = 47) during baseline (*A*, epoch “1avg10” from Fig. 1), fentanyl-induced apnea (*B*, *epoch 5* from Fig. 1), and naloxone recovery (*C*, epoch “8avg10” from Fig. 1). Each row represents a single unit from an individual experiment (one unit/experiment). Rows were divided into the inspiratory (*top*; unit Nos. 1–23) and expiratory (*bottom*; unit Nos. 24–47) classes. Row number corresponds to unit No. in Figs. 2 and 3. Inspiration was divided into 20 consecutive fractional bins (1–20) and expiration was divided into 40 consecutive fractional bins (21–60). Only the expiratory phase was sampled during apnea (*B*). *Z*-axis scale (*bottom left*) indicates average firing frequency per bin for 10 consecutive breaths. *A*–*C*, *right*: average firing frequency per bin of all units in the inspiratory class (*top*) or expiratory class (*bottom*). Line and error are means ± SE. Expiration was duplicated to display phase transitions. *D*: baseline-subtracted difference plot for firing frequency per bin in naloxone for individual units (*left*, *Z*-axis scale at *bottom right*) or averaged among inspiratory (*top*) or expiratory (*bottom*) class neurons (*right*). *D*, *right*: bins with significant differences from zero are shown in blue (inspiratory class) and red (expiratory class). *P < 0.05, by one-sample Wilcoxon signed-rank test.

**Figure 6.**
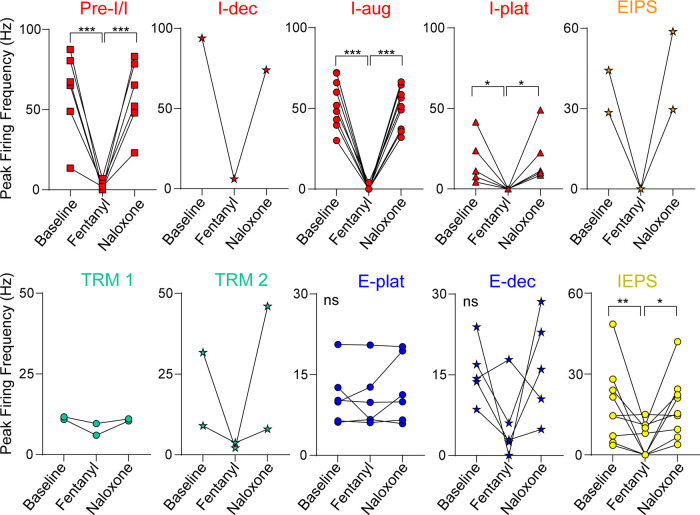
Firing frequency of dorsolateral pontine units during baseline, fentanyl-induced apnea, and naloxone recovery. Summary of single unit peak firing frequency at baseline, during fentanyl-induced apnea, and after fentanyl recovery. Peak firing frequency at baseline and after recovery with naloxone was averaged from 10 consecutive breaths. Peak firing frequency at apnea was determined during the duration of apnea (≥30 s). Units were placed into subgroups based on discharge identity. preinspiratory/inspiratory (pre-I/I; *n* = 6, red square), inspiratory augmenting (I-aug; *n* = 9, red circle), inspiratory plateau (I-plat; *n* = 5, red triangle), inspiratory/expiratory phase-S (IEPS; *n* = 9, yellow circle), expiratory plateau (E-plat; *n* = 6, blue circle), and expiratory decrementing (E-dec; *n* = 5, blue star) were statistically compared. Inspiratory decrementing (I-dec; n = 1, red star), expiratory/inspiratory phase-spanning (EIPS) (*n* = 2 orange star), tonic respiratory modulated expressing decreased activity during postinspiration (TRM1; *n* = 2, blue green circle), and tonic respiratory modulated expressing an increase in activity at inspiratory/expiratory phase transition (TRM2; *n* = 2, blue-green star) groups had too few *n*’s for statistical analysis. Symbols are single unit means from individual experiments. *P < 0.05, **P < 0.01, ***P < 0.001, ns = *P* > 0.05 for all comparisons by repeated-measures one-way ANOVA and Tukey’s post hoc test.

Inspiratory neuron activity was significantly reduced by fentanyl and most neurons in the inspiratory class fired zero action potentials during fentanyl-induced apnea (18 of 23, 78%) (Fig. 5). Among the inspiratory subgroups, I-aug units and pre-I/I units had significantly decreased firing frequency during apnea compared with baseline (Aug-I mean difference = 53.2 ± 5.0 Hz, *P* < 0.0001, *n* = 9; pre-I/I mean difference = 58.3 ± 10.9 Hz, *P* < 0.0001, *n* = 6; Fig. 6). All I-plat units were silent during fentanyl-induced apnea (5 of 5, mean difference = 17.6 ± 6.8 Hz, *P* = 0.0444; Fig. 6). In certain inspiratory subgroups, the sample size was insufficient for statistical analysis of grouped firing frequency data [I-dec (*n* = 1) and EIPS (*n* = 2)]. However, decreases in firing frequency were qualitatively obvious with both EIPS units silent during fentanyl-induced apnea (Fig. 6, I-dec and EIPS). Taken together, the majority of dorsolateral pontine inspiratory neurons were completely silent during fentanyl-induced apnea.

In contrast, most expiratory neurons (19 of 24, 79%) continued to fire throughout fentanyl-induced apnea, although often at a slower rate (Fig. 5). Among the expiratory subgroups, all E-plat units (*n* = 6) remained active during fentanyl-induced apnea with no change in firing frequency (Fig. 6). The majority of E-dec units (4 of 5) were active during fentanyl-induced apnea, and although qualitative decreases in firing frequency were observed in 4 of 5 neurons during fentanyl-induced apnea compared with baseline, the grouped data were not significant (mean difference = 9.6 ± 4.6 Hz, *P* = 0.1493; Fig. 6). TRM units also generally fired at a qualitatively lower frequency during fentanyl-induced apnea but nonetheless continued to fire (mean difference = 3.5 ± 1.4 Hz; 4 of 4, Fig. 6). Sample size was insufficient for statistical analysis of TRM group data [TRM1 (*n* = 2) and TRM2 (*n* = 2)]. IEPS units fired significantly slower during fentanyl-induced apnea (mean difference = 12.4 ± 3.5 Hz, *P* = 0.0081; Fig. 6) but the group showed mixed responses with a subset of the population not firing (4 of 9) and the other subset (5 of 9) continuing to fire. Taken together, most dorsolateral pontine expiratory neurons were relatively unaffected by fentanyl administration and continued to fire as if the apneic period was a prolonged expiration.

#### Naloxone recovery.

Fentanyl-induced apnea was reversed by the addition of the opioid antagonist naloxone (1 µM) to the perfusate. Respiration transitioned back to a three-phase pattern, but with shorter inspiratory and expiratory durations (Fig. 1*C*) resulting in a slightly faster rate (16.7 ± 5.3 breaths/min) when compared with baseline (mean difference = 4.4 ± 0.4 breaths/min, *P* < 0.0001).

To identify neuronal activity changes after naloxone reversal, the baseline heatmap (Fig. 5*A*) was subtracted from the naloxone heatmap (Fig. 5*C*) and corresponding bins were assessed for changes in firing frequency (Fig. 5*D*). Overall, the activity of the inspiratory class of neurons was significantly increased during pre-I (last 12.5% of the expiratory phase, bins 56–60, *P* < 0.05, Fig. 5*D*, inspiratory) and during early-inspiration (first 35% of the inspiratory phase, bins 1–7, *P* < 0.05) (*n* = 23). In contrast, there were no major changes in expiratory neuronal activity after naloxone reversal compared with baseline (Fig. 5*D*, expiratory). Thus, the shorter expiratory duration after naloxone is more likely related to an increase in pre-I/early-I activity, rather than the activity of expiratory neurons.

### Inspiratory Neuron Activity during Transitional Respiratory States

**Figure 7.**
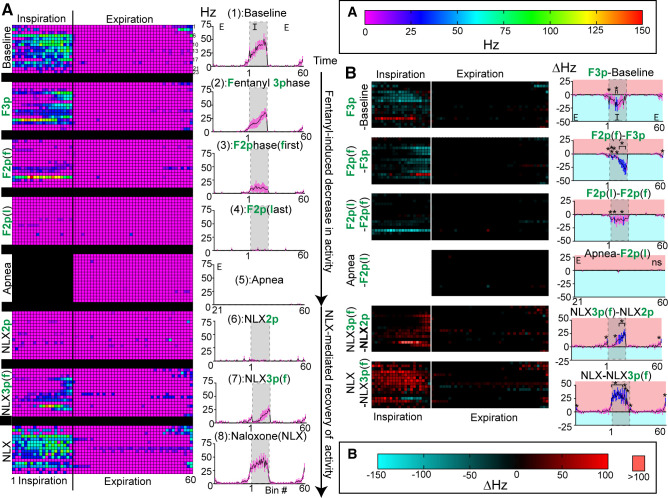
Activity of inspiratory units during fentanyl-mediated decline and naloxone-mediated recovery. *A*: phase fraction histograms of inspiratory class single unit activity from individual experiments (one unit/row, *left column*), or average activity of all inspiratory units (*right column*) at transitional epochs during fentanyl-mediated decline and naloxone-mediated recovery. Line and error are means ± SE. As depicted in Fig. 1. epochs included: (1) Baseline, (2, F3p) the last three-phase breath during fentanyl, [3, F2p(f)] the first two-phase breath during fentanyl, [4, F2p(l)] the last two-phase breath before apnea, (5) fentanyl-induced apnea, (6, NLX2p) the last two-phase breath during naloxone, [7, NLX3p(f)] the first three-phase breath during naloxone, and (8, NLX) a fully recovered breath post naloxone administration. *Z*-axis scale for left side of *A* is shown at top of right column. *B*: consecutive heatmaps were subtracted. Difference heatmaps display differences within individual experiments on each row (*B*, *left*) and difference histograms show differences in average activity (*B, right*). *B*, *right*: line and error are means ± SE. Bins with significant differences from zero are shown in blue. *P < 0.05, by one-sample Wilcoxon signed-rank test.

**Figure 8.**
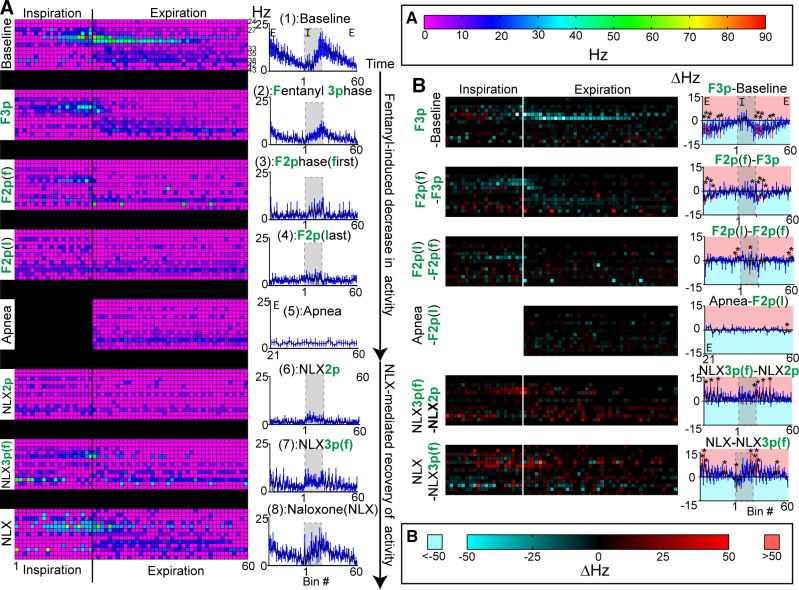
Activity of expiratory units during fentanyl-mediated decline and naloxone-mediated recovery. *A*: phase fraction two-dimensional (2-D) histogram (heatmap) of expiratory class single unit activity from individual experiments (one unit/row, *left column*) or average activity of all expiratory units (*right column*) at transitional epochs during fentanyl-mediated decline and naloxone-mediated recovery. Line and error are means ± SE. *Z*-axis scale for *A* (*left*) is at the top of right column. As depicted in Fig. 1. epochs included: (1) Baseline, (2, F3p) the last three-phase breath during fentanyl, [3, F2p(f)] the first two-phase breath during fentanyl, [4, F2p(l)] the last two-phase breath before apnea, (5) fentanyl-induced apnea, (6, NLX2p) the last two-phase breath during naloxone, [7, NLX3p(f)] the first three-phase breath during naloxone, and (8, NLX) a fully recovered breath post naloxone administration. *B*: consecutive heatmaps were subtracted. Difference heatmaps display differences with individual experiments on each row (*B*, *left*) and difference histograms show differences in average activity (*B*, *right*). *B*, *right*: line and error are means ± SE. Bins with significant differences from zero are shown in red. *P < 0.05, by one-sample Wilcoxon signed-rank test.

The “static comparison” of neural activity during fentanyl-induced apnea and after naloxone recovery does not take into account the dynamic changes that occur during the transitions between breathing states. To investigate how pontine neuronal activity changed at these transitions, single neuronal unit activity was analyzed at each of the eight epochs described in Fig. 1. These are shown for each inspiratory (Fig. 7*A*) and expiratory unit (Fig. 8*A*) and differences were determined by subtraction of the histograms, as indicated (Fig. 7*B*and Fig. 8*B*). Only experiments that contained all eight epochs were used for this analysis (*n* = 29).

During the slowed three-phase breath after fentanyl (F3p), units in the inspiratory class fired significantly less on average in the middle of inspiration (35%–50% of inspiration, bins 8–10, *P* < 0.05) as compared with baseline (Fig. 7*B*, F3p-Baseline). Therefore, as inspiratory duration increased in the three-phase state with fentanyl perfusion, dorsolateral pontine inspiratory neuronal activity occurred less in the middle of the breath.

As the respiratory output progressed to the first two-phase breath (F2p(f)), there was a significant loss of inspiratory neuronal activity during the late-inspiratory period (50%–95% of inspiration, bins 11–19, *P* < 0.05), compared with the preceding three-phase breath (Fig. 7*B*, F2p(f)-F3p). Thus, as the system transitioned from the three-phase to the two-phase pattern, late-inspiratory dorsolateral pontine neuronal activity was lost.

During the last two-phase breath before apnea (F2p(l)), nearly all the remaining inspiratory neuronal activity stopped (Fig. 7*A*, F2p(l)) with significant decreases during select bins in inspiration (bins 1, 5, 15, *P* < 0.05), as compared with the first fentanyl-induced low amplitude breath (Fig. 7*B*, F2p(l)-F2p(f)). However, this significant decrease stemmed from decreased activity of only a few neurons.

To compare activity from the last two-phase breath to apnea, only the expiratory phase of the histogram was subtracted, since there is no inspiratory phase during apnea. During apnea, there were no statistically significant changes in firing frequency among the inspiratory class, as compared with the expiratory period during the last fentanyl-induced low amplitude breath (Fig. 7*B*, Apnea-F2p(l), *P* > 0.05). Thus, dorsolateral pontine inspiratory neuronal activity in the last 2-phase breath is similar to fentanyl-induced apnea, with both having low levels of inspiratory neuronal activity.

After sustained apnea (≥30 s), naloxone was added to reverse fentanyl’s effects. Phrenic output emerged from apnea as fast, low amplitude, long duration bursts (Fig. 1*B*, *epoch 6*) before abruptly transitioning to a three-phase respiratory pattern wherein the first three-phase breath had a very long inspiratory duration (Ti = 3.2 ± 1.9 s, Fig. 1, *B*and *C*, *epoch 7*). During the first three-phase breath after naloxone (NLX3p), inspiratory neurons fired significantly more mainly during the late-inspiratory period (60%–100% of inspiration, bins 10, 12–20, 60, *P* < 0.05) compared with the preceding two-phase breath (Fig. 7*B*, NLX3p-NLX2p). Taken together, recovery was similar to the decline, in that late-inspiratory dorsolateral pontine neuronal activity occurred only in normally patterned breaths (3-phase) and not in low amplitude breaths (2-phase).

Once breathing stabilized, inspiratory duration was shorter (Ti = 0.68 ± 0.02 s, Fig. 1*C*, *epoch 8*) and inspiratory activity was compared from the first three-phase breath to a three-phase breath after full recovery with naloxone. The inspiratory class fired significantly more during most of the inspiratory phase following full recovery (first 60% of inspiration, bins 1–12, 14, 15, 17, 60, *P* < 0.05) as compared with the first three-phase breath post naloxone (Fig. 7*B*, NLX-NLX3p).

### Expiratory Neuron Activity during Transitional Respiratory States

Expiratory neuron activity was analyzed at the same transitional state epochs as aforementioned. During the slowed three-phase breath in fentanyl (F3p), neurons in the expiratory class fired significantly less during the early-expiratory period (bins 22, 24, 27–28, 37, 41, Fig. 8*B*, *P* < 0.05), as compared with baseline (Fig. 8*B*, F3p-Baseline). During the first two-phase breath, the expiratory class on average fired significantly less before and after the inspiratory/expiratory phase transition (bin 16, 23–24, 29, 31, *P* < 0.05), as compared with the prior breath (Fig. 8*B*, F2p(f)-F3p). This loss of late-inspiratory/postinspiratory neuronal activity was not surprising as postinspiratory vagal output is also absent in the two-phase state (Fig. 1*B*, *epoch 3*). Expiratory neuronal activity was generally unchanged during further decline in output from the two-phase state and was mainly tonic (lack of phase relationship) in the two-phase state and apnea (Fig. 8*B*, F2p(l)-F2p(f), Apnea-F2p(l)).

Naloxone-mediated recovery of expiratory neuronal activity was similar to the manner in which it was depressed. During the first 3-phase breath after naloxone, the expiratory class fired significantly more during late-inspiration (bin 19) and early-expiration (bin 22, 28, 36), as compared with the prior two-phase breath (Fig. 8*B*, NLX3p(f)-NLX2p). During the fully recovered three-phase breath, the expiratory class fired significantly more during late-inspiration (bin 18), early-expiration (bin 23, 25, 26), and late-expiration (48), as compared with the first three-phase breath post naloxone administration (Fig. 8*B*, NLX-NLX3p).

**Figure 9.**
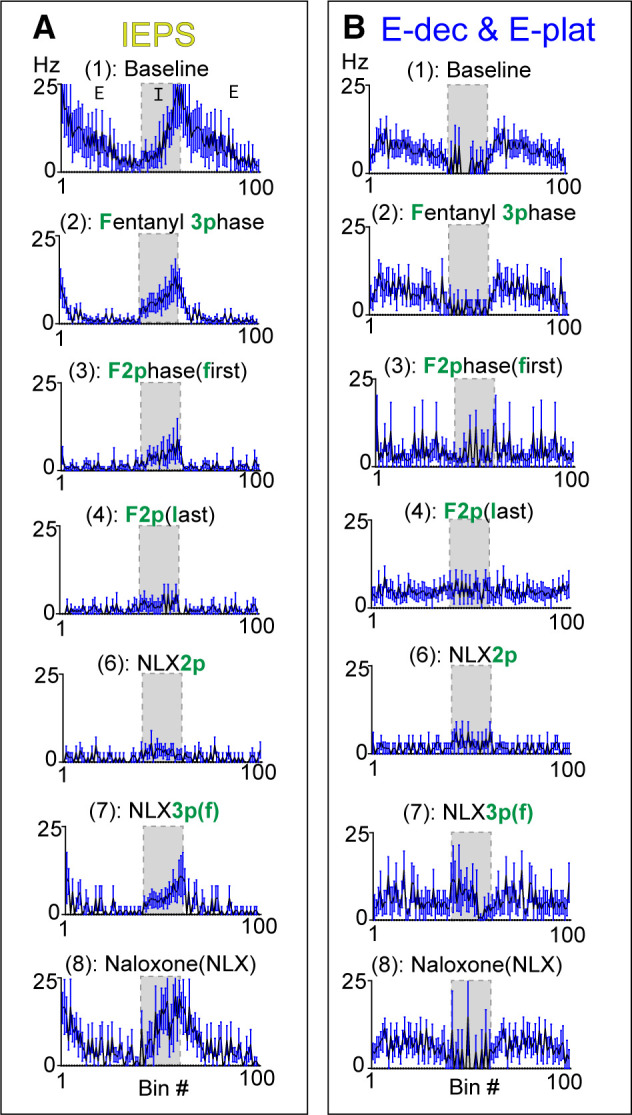
Activity of expiratory class subgroups during fentanyl-mediated decline and naloxone-mediated recovery. The expiratory class was divided into two groups: inspiratory/expiratory phase-spanning (IEPS) (*A*, *n* = 7) and expiratory decrementing (E-dec)/ expiratory plateau (E-plat) (*B*, *n* = 7; 3 E-dec, 4 E-plat). As depicted in Fig. 1, epochs included: (1) Baseline, (2, F3p) the last three-phase breath during fentanyl, [3, F2p(f)] the first two-phase breath during fentanyl, [4, F2p(l)] the last two-phase breath before apnea, (5) fentanyl-induced apnea, (6, NLX2p) the last two-phase breath during naloxone, [7, NLX3p(f)] the first thre-phase breath during naloxone, and (8, NLX) a fully recovered breath post naloxone administration.

In the presence of fentanyl, units among the expiratory class had somewhat opposing changes in activity during the inspiratory phase. This was not captured in the expiratory class summary, so we also separately analyzed the activity of two subgroups of expiratory neurons: *1*) IEPS (*n* = 7) and *2*) E-dec/E-plat (*n* = 7) (Fig. 9). In the last three-phase breath and the first two-phase breath, IEPS activity was expressed mainly in the inspiratory phase, and not in the postinspiratory phase. In the E-dec/E-plat group, neuronal activity was quiescent during inspiration at baseline. This quiescence of inspiratory related activity was lost during two-phase breaths, transforming the activity of these E-dec/E-plat neurons from phasic to tonic.

### Location of Recorded Units

Histology revealed that recording locations were clustered in and surrounding the Kölliker–Fuse nucleus and lateral parabrachial complex with some recordings just dorsal or ventral to the target. Inspiratory and expiratory neurons appear to be co-mingled throughout the dorsolateral pons with clustering in the Kölliker–Fuse/parabrachial complex and supratrigeminal region (Fig. 4*B*). This co-mingling of inspiratory and expiratory pontine neurons has been previously described (27–32), however a more complex anatomical model with interweaving local populations has also been proposed (33).

### Assessing Alternative Influence

To assure that fentanyl-induced changes in respiratory pattern and dorsolateral pontine neuron firing activity were the results of drug administration as opposed to alterations in perfusion pressure, pH, or oxygen availability, in a subset of experiments, tissue pH and oxygen levels within the dorsolateral pons were analyzed at baseline, during fentanyl-mediated apnea, and 2 min after naloxone reversal. Tissue oxygen levels and pH were unchanged by fentanyl and naloxone (O_2_: baseline = 134 ± 4 mmHg, fentanyl = 135 ± 4 mmHg, NLX = 142 ± 6 mmHg, *P* > 0.05 for all comparisons by repeated-measures one-way ANOVA and Tukey’s posttest, *n* = 4), (pH: baseline = 7.13 ± 0.03, fentanyl = 7.14 ± 0.03, NLX = 7.15 ± 0.03, *P* > 0.05 for all comparisons by repeated-measures one-way ANOVA and Tukey’s posttest, *n* = 5). Perfusion pressure was also unchanged by fentanyl perfusion, but slightly increased by naloxone [baseline = 64 ± 3 mmHg, fentanyl = 64 ± 3 mmHg (*P* > 0.05 compared with baseline, *n* = 15) NLX = 66 ± 3 mmHg (*P* < 0.001 compared with baseline by repeated-measures one-way ANOVA and Tukey’s posttest, *n* = 15)]. Thus, fentanyl-mediated changes in dorsolateral pontine neuronal activity are not due to changes in perfusion pressure or local changes in pH or oxygen levels.

### Identification and Opioid Sensitivity of Swallow-Associated Dorsolateral Pontine Units

**Figure 10.**
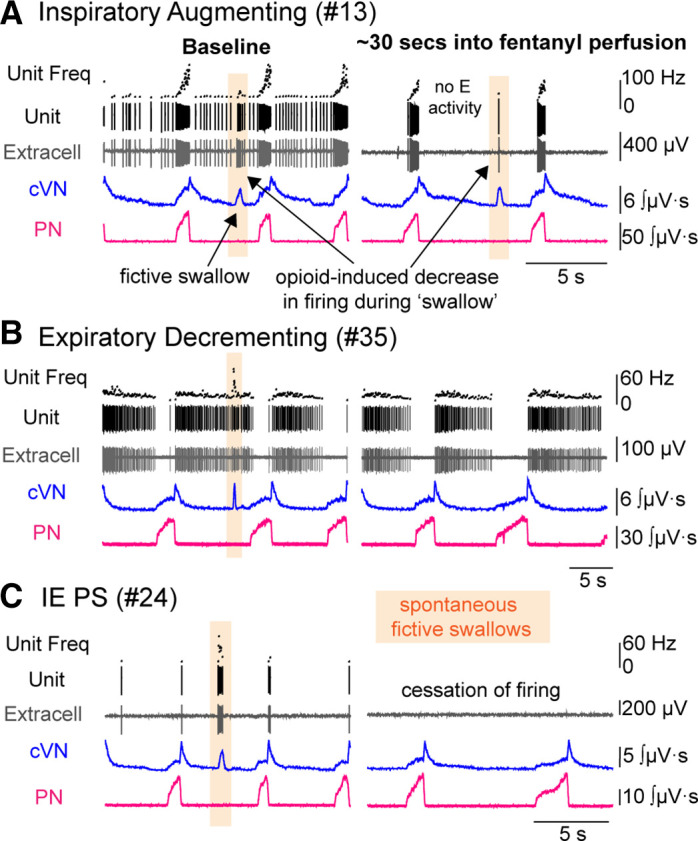
Swallow-associated dorsolateral pontine neurons. Recordings from central vagus nerve (cVN), phrenic nerve (PN), and single unit activity in the dorsolateral pons were made using an in situ preparation of rat. Example traces for three experiments demonstrating dorsolateral pontine neurons involved with fictive breathing and swallowing are shown at baseline (*left*) and ∼30 s after perfusion of fentanyl (300 nM) (*right*). Dorsolateral pontine inspiratory (*A*), expiratory decrementing (*B*), and inspiratory/expiratory phase-spanning (IEPS, *C*) respiratory patterned neurons burst with spontaneous fictive swallows (orange highlight) at baseline. After fentanyl perfusion (300 nM), the IEPS neuron (*C*) ceases to fire. At the same time, the inspiratory neuron (*A*) and expiratory neuron (*B*) are still firing. Note in *A*, there is decreased firing of the inspiratory neuron during the swallow in fentanyl compared to the swallow at baseline.

In addition to rhythmic respiratory-related output, we observed in 91% of experiments short duration vagal bursts that occurred during the expiratory phase of three-phase breaths (Fig. 10), akin to spontaneous fictive swallows that have been previously described (34). These spontaneous vagal bursts were infrequent, occurring sometimes only once or twice during a recording, and were not always captured during the duration of the extracellular unit recording (∼30 min). However, in 37 experiments these bursts did occur during extracellular unit recording, and 27% of units (10 out of 37 units) displayed changes in firing frequency during spontaneous fictive swallow (Fig. 10). Thus, these units had multifunctional activity during both fictive swallowing and breathing. The respiratory pattern of these swallow-associated units was diverse, but tended to be expiratory associated (80%), and were observed in the following subgroups: IEPS (3 of 10 units), E-dec (3 of 10 units), E-plat: 1 of 10 units, TRM2: 1 of 10 units, I-aug (1 of 10 units), Pre-I/I (1 o of 10 units). Only four of these swallow-associated units were silent during fentanyl-induced apnea (1 I-aug, 2 IEPS, and 1 E-dec). This was not surprising considering most of the swallow-associated units were expiratory and the majority of pontine expiratory units were active during fentanyl-induced apnea (Fig. 5). Interestingly, swallow-associated neurons were located exclusively in the Kölliker–Fuse and neighboring supratrigeminal region (Fig. 3*B*).

## DISCUSSION

This study identified several categories of dorsolateral pontine neurons based on phasic respiratory-associated discharge patterns, which were differentially affected by fentanyl. The major findings were that dorsolateral pontine inspiratory neurons were largely silenced by fentanyl administration, whereas expiratory neurons were not. The silencing of inspiratory neurons observed in the pons is similar to the opioid-sensitivity of inspiratory neurons in the pre-Bötzinger complex (preBötC), which are inhibited by opioids (22, 35, 36) through both pre- and postsynaptic mechanisms (23). However, preBötC expiratory neurons are also inhibited by opioids. Expiratory neurons in the ventrolateral medulla, including the preBötC, are silenced by apneic concentrations of fentanyl (22), and opto-tagging showed that half of the preBötC expiratory neurons express μ-opioid receptors (23). In the dorsolateral pons, only 21% of expiratory neurons were silenced during fentanyl-induced apnea. We propose that the pontine expiratory neurons that continue to fire tonically during fentanyl administration could provide tonic drive to expiratory neurons in the medulla and promote apnea.

In addition, we tracked the activity of dorsolateral pontine neurons during the fentanyl-induced transition to apnea and the naloxone-mediated transition to recovery. We found that the patterning of dorsolateral pontine inspiratory and expiratory class activity closely mirrored phrenic and vagal motor nerve output, respectively, at baseline and during all opioid receptor-mediated output patterns. Most notably, late-inspiratory and postinspiratory neuronal activity was lost during the transition to two-phase output. In fact, late-inspiratory and postinspiratory dorsolateral pontine neuronal activity always occurred in the three-phase output and never occurred during shallow two-phase respiratory output. This finding suggests pontine neuronal activity is correlated with three-phase breathing (defined by postinspiratory vagal output and augmenting phrenic output), which is highly congruent with the role of the dorsolateral pons in the inspiratory off-switch (17, 19, 20, 37–40), and the loss of decrementing vagal postinspiratory activity and augmenting phrenic inspiratory activity following opioid administration into the dorsolateral pons (11).

### Cellular Mechanisms of Opioid Inhibition

Opioids could inhibit neuronal firing activity through both pre- and postsynaptic mechanisms. μ-Opioid receptor expression in the lateral parabrachial area is extremely abundant (13, 41, 42). Lateral parabrachial and KF neurons are hyperpolarized by μ-opioid receptor activation of G protein-coupled potassium conductance (11, 43), including neurons that project to the preBötC and rostral ventral respiratory group (rVRG) (44). Thus, we suspect that inspiratory and a subset of IEPS neurons that are completely silent during fentanyl-induced apnea could be hyperpolarized by activation of postsynaptic μ-opioid receptors but cannot rule out presynaptic mechanisms. In contrast, the majority (79%) of expiratory neurons lost their phasic respiratory patterning but continued to fire during fentanyl-induced apnea, likely indicating a loss of presynaptic inputs. For E-dec/E-plat neurons, inspiratory quiescence was lost, suggesting a loss of inspiratory phase inhibition. This inspiratory phase inhibition could be coming from inhibitory preBötC neurons, which project to the pons (45), express μ-opioid receptors (12), and/or connect to pontine expiratory neurons (46).

μ-Opioid receptors are broadly expressed in glutamatergic, glycinergic, and GABAergic preBötC neurons (12). The neurotransmitter phenotype of the dorsolateral pontine neurons we recorded is unknown. The dorsolateral pons contains segregated populations of glutamatergic and GABAergic neurons (47). Glutamatergic dorsolateral pontine neurons project to respiratory-related areas of the ventrolateral medulla (47) and express μ-opioid receptors (41, 44). We predict that the opioid-sensitive inspiratory neurons are glutamatergic, but this remains to be determined.

### Discharge Identity of Dorsolateral Pontine Neurons

The discharge identities of the neurons recorded in this study were consistent with previous literature and our expectations, with a few exceptions that are outlined later. We classified neurons into subgroups based on observed activity and previously established pattern-naming schemes for populations of respiratory neurons (27, 48). In our study, 30% of units had an augmenting discharge during inspiration (including pre-I/I and I-aug, but excluding IEPS). This is a slightly higher proportion than what has been previously reported using the arterially perfused preparation [22% units in “other” group containing I-aug, E-plat, and inspiratory modulated (40), but slightly lower than studies using vagotomized, decerebrate, ventilated cats (36% of respiratory modulated neurons in the pons had an I-aug discharge (27)]. In addition, the highest frequency of any dorsolateral pontine activity occurred in late inspiration, which is representative of inspiratory/postinspiratory local field potentials (LFPs) that have been demonstrated in the pontine respiratory group (37), since neuronal bursts can predict LFP features (49).

In our study, 19% of units had an IEPS discharge. This is similar to the 22% reported previously using the arterially perfused preparation (40). In contrast, only 11% of recorded units had an E-dec discharge, compared with the 39% previously reported (40). Although augmenting expiratory (aug-E) neurons have been observed in the dorsolateral pons of anesthetized, ventilated rat preparations (31, 32) and decerebrate, vagotomized, ventilated cats (27), they were not observed in our experiments. Dorsolateral pontine aug-E neurons potentially play a role in the formation of late-E abdominal nerve activity (50). In the arterially perfused preparation, late-E abdominal nerve bursting is absent in normocapnic conditions [5% CO_2_ (51)]. Therefore, late-E abdominal drive was low in our experiments and potentially as a result, pontine aug-E neuronal firing was not observed. This is consistent with the lack of aug-E patterned neurons reported in the pons under normocapnic conditions using the arterially perfused preparation (40).

We did not include any nonrespiratory modulated neurons, even though the tonic pontine drive to medullary respiratory neurons plays an important role in models of respiratory pattern formation (46, 48, 52, 53). The dorsolateral pons, especially the lateral parabrachial area, has abundant expression of μ-opioid receptor-containing neurons that are involved in behaviors other than breathing, such as taste, fear, and pain (54–56). Our aim was to describe the effects of fentanyl on respiratory-related neuronal activity, so we excluded tonic neurons since we could not verify their role in respiration. In addition, respiratory modulated neurons were frequently encountered, since the in situ preparation lacks vagal input, consistent with the expected increase in respiratory-related activity in the dorsolateral pons with removal of vagal input (27, 29, 48).

### Pontine Late-Inspiratory Ramping Activity

The highest frequency neuronal activity we observed at baseline among all pontine units occurred during late inspiration and was absent during fentanyl-induced low amplitude phrenic output. The late-inspiratory pontine activity could be mediated by excitatory drive from I-aug medullary neurons (46, 52), decrementing inhibition from I-dec medullary neurons (46) or possibly from I-dec pontine neurons (40, 57). Pontine I-dec neurons were rare in our data set likely due to sampling (*n* = 1 I-dec and *n* = 2 EIPS), but they have been reported by others (40, 57). Intrapontine connections have been documented (52), proposed to be involved in the inspiratory off-switch mechanism (40), and supported by computational models of the respiratory network (48, 58). One of these intrapontine connections was an inhibitory connection between an I to IE intrapontine pair (52), suggesting decrementing inhibition during inspiration could exist within the pons, but further investigation of the nature and extent of such connections is warranted.

It is unclear whether the loss of pontine late-I activity directly causes the change in phrenic output, but the pathways exist for it to be involved. Inspiratory neurons in the dorsolateral pons project to the ventrolateral medulla (31), and excitatory Kölliker–Fuse neurons project to the rVRG and phrenic motor pool (47, 59), which contain augmenting inspiratory phrenic premotor and motor neurons, respectively. Pharmacological inhibition of the dorsolateral pons leads to decreases in late-inspiratory activity in medullary neurons (60) suggesting pontine late-inspiratory drive contributes to medullary late-inspiratory activity. According to the model proposed by Mörschel and Dutschmann (40), it could be that loss of excitatory I-aug input from the medulla causes loss of pontine late-I activity, which then eliminates descending excitatory input from the pontine late-I neurons to inhibitory medullary late-I neurons that initiate the inspiratory off-switch (29), thereby delaying inspiratory termination and leading to changes in the phrenic output pattern.

Fentanyl can disrupt this pathway in multiple ways. First, medullary propriobulbar inspiratory neurons are inhibited by fentanyl (22). Second, opioids inhibit dorsolateral pontine neurons (11) that provide excitatory input on preBötC and rVRG neurons (44). Finally, activation of opioid receptors in the dorsolateral pons eliminates the ramping phrenic output pattern and causes apneusis (11).

### PreBötC and Dorsolateral Pontine Interdependent Mechanisms

The dorsolateral pons and preBötC are highly interconnected (45, 47, 48, 52, 61), and rhythmic pontine inspiratory activity is dependent upon inputs from the medulla [“efference copy” (27, 29, 62)]. Opioids slow inspiratory rhythmogenesis within the preBötC via mechanisms requiring μ-opioid receptor expression in Dbx1-expressing neurons (12, 63). Thus, the effects of fentanyl we observed could be mediated, at least in part, by the activation of opioid receptors in the medulla. For instance, opioid inhibition of propriobulbar inspiratory neurons could prolong inspiratory time (22).

Inhibition of I-Aug medullary neurons that project to the pons could lead to the inhibition of postinspiratory pontine neuronal activity (40, 48, 52). Opioid inhibition of inhibitory, inspiratory preBötC neurons that project to pontine expiratory neurons could inhibit inspiratory quiescence leading to the tonic firing of pontine expiratory neurons (12, 45, 46). Given that medullary activity is sufficient to produce rhythmic two-phase output (64), the low-amplitude two-phase bursts that occurred after fentanyl administration and in the absence of pontine inspiratory activity are possibly due to medullary rhythmogenic activity that persists even after inspiratory activity in the pons is lost following opioid administration.

### Opioid Effects on Dorsolateral Pontine Involvement in Upper Airway Control

Upper airway and breathing coordination diminish in the presence of opioids leading to difficulty swallowing, aspiration, and sleep-disordered breathing in patients (65–67). This coordination is partly controlled by the dorsolateral pons, as lesion of dorsolateral pontine Kӧlliker–Fuse/parabrachial complex impairs the ability of the respiratory network to accommodate a swallow (68). Furthermore, the KF has been suggested to mediate the laryngeal adductor reflex, which protects the airway from aspiration (34). Dorsolateral pontine neurons have one-to-many axon collateralization (61) with projections to multiple respiratory and upper airway nuclei and motor pools, including preBötC, BötC, nucleus of the solitary tract, and hypoglossal, facial, and phrenic motor nuclei (31, 47, 61, 69), and inputs from medullary respiratory control neurons (45, 46, 52), which could facilitate this coordination. We observed a subset of dorsolateral pontine neurons that changed firing frequency during both breathing and fictive swallowing, suggesting they could be involved in the coordination of breathing and upper airway control. The swallow-associated neurons we recorded were localized to the Kölliker–Fuse/supratrigeminal region, overlapping the population of FoxP2 expressing dorsolateral pontine neurons that are ventral to the external lateral parabrachial area in rats (47, 70, 71). A subset (40%) of these swallow-associated neurons was silent during fentanyl-induced apnea that could contribute to opioid-induced upper airway dysfunction. In addition, since some inspiratory KF neurons project to the hypoglossal motor nucleus (31), some of the inspiratory neurons inhibited by fentanyl could be hypoglossal premotor neurons, or otherwise involved in the coordination of hypoglossal activity.

Breathing and swallowing use overlapping muscles, although not to the same extent during different behaviors i.e., glottal adduction is more pronounced during swallowing (laryngeal closure) than breathing (expiratory airflow “braking”) (72). Accordingly, an IEPS neuron that was silent during fentanyl-induced apnea was weakly active at the IE respiratory phase transition but strongly active during fictive swallows (Fig. 10*C*). Thus, neurons such as this IEPS neuron may play a more dominant role in swallowing than breathing, or potentially the laryngeal adductor reflex than breathing.

### Conclusions

In conclusion, dorsolateral pontine inspiratory neuronal activity was significantly decreased during fentanyl-induced apnea, with most inspiratory neurons completely silent. The majority of expiratory neurons were active during fentanyl-induced apnea but fired in a tonic pattern. Late-inspiratory and postinspiratory pontine neuronal activity was lost as the system transitioned to a low amplitude two-phase state. The loss of dorsolateral pontine activity during fictive fentanyl overdose could mediate pontine-mediated opioid-induced respiratory and upper airway deficits. Together, these data further our understanding of the neuronal mechanisms underlying the deleterious effects of opioids on breathing.

## GRANTS

This work was supported by National Institutes of Health Grant R01DA047978.

## DISCLOSURES

No conflicts of interest, financial or otherwise, are declared by the authors.

## AUTHOR CONTRIBUTIONS

S.E.S. and E.S.L. conceived and designed research; S.E.S. and D.M.B. performed experiments; S.E.S. and D.M.B. analyzed data; S.E.S., D.M.B., and E.S.L. interpreted results of experiments; S.E.S. prepared figures; S.E.S. and E.S.L. drafted manuscript; S.E.S., D.M.B., and E.S.L. edited and revised manuscript; S.E.S., D.M.B., and E.S.L. approved final version of manuscript.
